# Peroxisomal abnormalities and catalase deficiency in Hutchinson-Gilford Progeria Syndrome

**DOI:** 10.18632/aging.102941

**Published:** 2020-03-18

**Authors:** Xiaojing Mao, Pratima Bharti, Abhirami Thaivalappil, Kan Cao

**Affiliations:** 1 Department of Cell Biology and Molecular Genetics, College of Computer, Mathematical, and Natural Sciences, University of Maryland, College Park, MD 20742, USA

**Keywords:** peroxisome, progeria, catalase, ROS, rapamycin

## Abstract

Peroxisomes are small, membrane-enclosed eukaryotic organelles that house various enzymes with metabolic functions. One important feature in both Hutchinson-Gilford Progeria Syndrome (HGPS) and normal aging is the elevated levels of Reactive Oxygen Species (ROS), which are generated from metabolic pathways with the capacity to cause oxidative damage to macromolecules within the cells. Although peroxisomal bioreactions can generate free radicals as their byproducts, many metabolic enzymes within the peroxisomes play critical roles as ROS scavengers, in particular, catalase. Here, we observed impaired peroxisomes-targeting protein trafficking, which suggested that the poorly assembled peroxisomes might cause high oxidative stress, contributing to the premature senescent phenotype in HGPS. We then investigated the ROS clearance efficiency by peroxisomal enzymes and found a significantly decreased expression of catalase in HGPS. Furthermore, we evaluated the effects of two promising HGPS-treatment drugs Methylene Blue and RAD001 (Everolimus, a rapamycin analog) on catalase in HGPS fibroblasts. We found that both drugs effectively reduced cellular ROS levels. MB, as a well-known antioxidant, did not affect catalase expression or activity. Interestingly, RAD001 treatment significantly upregulated catalase activity in HGPS cells. Our study presents the first characterization of peroxisomal function in HGPS and provides new insights into the cellular aspects of HGPS and the ongoing clinical trial.

## INTRODUCTION

Hutchinson-Gilford Progeria Syndrome (HGPS) is a devastating autosomal dominant genetic disorder affecting one in 4-8 million children worldwide [1, 2]. Characterized by a failure to thrive, loss of subcutaneous fat, alopecia, osteolysis, and scleroderma; children with this disorder have a markedly premature aging phenotype [3, 4]. The disorder is caused by a *de novo* point mutation in the exon 11 of the *LMNA* gene (c.1824C>T), whereby activation of a cryptic splice site deletes a 150 nucleotide sequence from exon 11, ultimately producing a truncated version of pre-lamin A that retains a toxic farnesyl modification, termed progerin [1, 5]. The amassing of aberrant protein at the nuclear envelope results in nuclear blebbing [5], defective DNA replication and repair [6, 7], alterations in chromatin organization [8], mitochondrial dysfunction [9, 10] and disruption of cellular redox homeostasis [11].

One defining feature in both HGPS and normal aging is the accumulated cellular oxidative stress, which occurs when there is an imbalance between the production of reactive oxygen species (ROS) and antioxidants that curb ROS initiation and propagation [12]. The mitochondria act as a major source of ROS through oxidative phosphorylation and other metabolic processes [10]. In HGPS, ROS levels become elevated due to mitochondrial dysfunction, as there is a distinct downregulation of mitochondrial oxidative phosphorylation proteins, morphological abnormalities, and lessened movement [9, 10]. Increased levels of ROS can lead to cellular and molecular damage in the body, which is why ROS scavengers in peroxisomes play a key role in the management of oxidative stress [13]. Peroxisomes are small, membrane-bound eukaryotic organelles that contain various enzymes to carry out metabolic reactions that convert ROS into hydrogen peroxide, which is then decomposed by ROS scavenging enzymes such as catalase or glutathione peroxidase (GPx) into water and oxygen [14, 15]. Peroxisomes biogenesis disorders, peroxisomal matrix proteins mis-localization and catalase deficiency have been reported in normal aging cells [16].

Many treatment strategies for HGPS have been proved to help reduce ROS production in the organism, including RAD001 and Methylene Blue (MB) [17]. RAD001 (or Everolimus) is a rapamycin analog that promotes the progerin clearance in HGPS cells by activating the autophagy machinery [18, 19]. It rescues many cellular defects in HGPS cells and is currently being used in clinical trials for HGPS patients [20, 21]. Methylene Blue (MB) is a mitochondrial-targeting antioxidant whose redox property allows it to cycle between reduced and oxidized forms thus facilitating electron transfer in the mitochondria. This reduces ROS production by the increase of mitochondrial oxidative phosphorylation and the reduction of electron leakage [22]. Our recent work with MB in HGPS demonstrates that the antioxidant rescues many aging signs of HGPS, such as nuclear blebbing and heterochromatin loss, and improves mitochondrial health [9].

The purpose of this study is to investigate the interplay between progerin, oxidative stress, and peroxisomes in HGPS cells. We characterized the peroxisomes in HGPS fibroblasts by investigating their assembly, protein import, and ROS scavenging enzyme activity. We also evaluated the effect of two promising HGPS-treatment drugs Methylene Blue and RAD001 on peroxisomes in HGPS cells.

## RESULTS

### Peroxisomal biogenesis in HGPS fibroblasts

**Figure 1:**
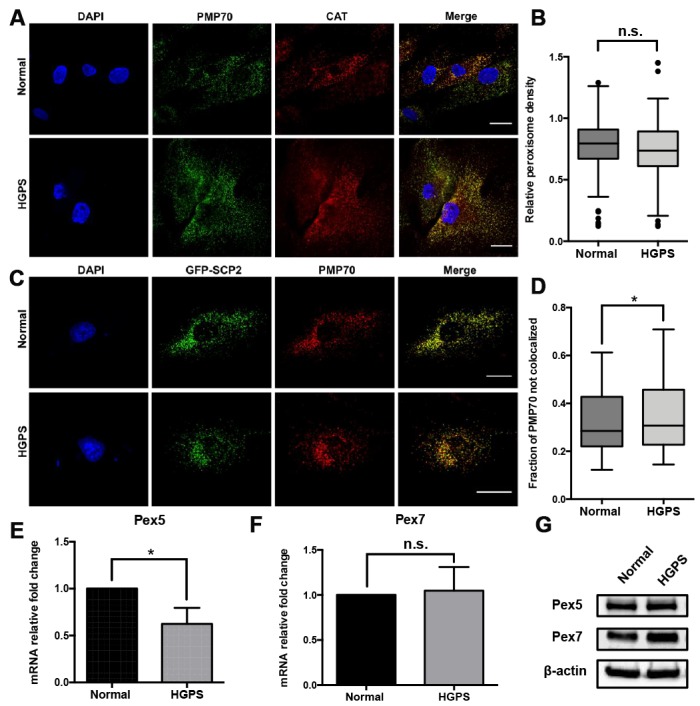
**Characterization of the peroxisomes in HGPS fibroblasts.** (**A**) Peroxisomes localization indicated by PMP70 and catalase immunofluorescence staining in HGPS patient-derived dermal fibroblasts and its normal counterpart. Bar = 25μm. (**B**) The peroxisomes density per cell was indicated by the quantification of PMP70 puncta per square unit in normal and HGPS fibroblast cells. More than 100 cells from 3 independent experiments were analyzed and the data was represented in Tukey box plot. Boxes show the 25^th^, 50^th^, and 75^th^ percentiles and the dots indicate the outliers. (**C**) Confocal fluorescence microscopy analysis of normal and HGPS fibroblasts transfected with GFP-SCP2, and probed with PMP70 antibody. Bar = 25μm. (**D**) Box plot of the portion of PMP70 puncta not colocalized with SCP2 (peroxisomal ghosts) in each cell. More than 100 cells from 3 independent experiments were analyzed. Boxes show the 25th, 50th, and 75th percentiles. Kolmogorov-Smirnov test was used to compare the distributions of these two samples. (**E**, **F**) Quantitative RT-PCR analysis of the relative expression of Pex5 and Pex7 in normal and HGPS fibroblasts. (**G**) Western blot analysis of Pex5 and Pex7 in normal and HGPS fibroblasts at passage 21. All experiments were performed using mid-passage cells between p15 to p25. All experiments were repeated at least three times and representative data were shown as indicated. *, p < 0.05, n.s., not significant.

To determine whether the extreme cellular environment in HGPS fibroblasts causes peroxisomal biogenesis disorder, we first detected the peroxisomal distribution by confocal fluorescence microscopy. Within the cytosol of each cell, the puncta co-stained with both 70-kDa peroxisomal membrane protein (PMP70) and catalase antibodies are identified as mature and functional peroxisomes (Figure 1A). We found that the majority of peroxisomes stained are mature and that there is no significant difference between the PMP70-catalase colocalizations in normal and HGPS fibroblasts (Supplementary Figure 1A). We then quantified the peroxisomes abundance in more than 200 fibroblast cells from normal and HGPS samples using PMP70 as a marker (Figure 1B), and found that compared to normal cells, there was no significant change in peroxisome density in HGPS cells.

In cells with peroxisomal biogenesis defects, aberrant peroxisomes lacking some of the matrix proteins were often observed, referred to as “peroxisome ghosts” [23]. To investigate the peroxisomal matrix proteins import machinery in HGPS fibroblasts, cells were transfected by vectors encoding GFP-tagged sterol carrier protein 2 (GFP-SCP2). SCP2 is a peroxisomal targeting protein with comparable expression levels between normal and HGPS fibroblasts (Supplementary Figure 1B and 1C). At 48 hours post-transfection, these cells were fixed and immunostained with an antibody against the peroxisomal membrane protein PMP70. The cellular distribution of GFP-SCP2 and PMP70 was detected by confocal fluorescence microscopy. We identified the SCP2-deficient “peroxisome ghosts” by a customized pipeline in CellProfiler [24], which quantified the portion of PMP70 puncta not co-localized with GFP-SCP2 in each cell. The results acquired from more than 100 cells indicated that compared to normal fibroblasts, there was a larger portion of HGPS cells with higher GFP-SCP2-deficient “peroxisome ghosts” index (Figure 1C, 1D).

In mammalian cells, the recognition and transportation of peroxisomal-targeting proteins were carried out by two cytosolic transporters Pex5 and Pex7 [25]. Quantitative RT-PCR revealed that the relative expression of Pex5, the receptor for peroxisomal targeting sequence 1 (PTS1)-containing proteins including catalase [26], and SCP2 [27], decreased slightly in HGPS fibroblasts (Figure 1E). However, this reduction was not detected in the Western blot assay (Figure 1G). Meanwhile, the expression of Pex7 remained unchanged in HGPS fibroblasts (Figure 1F and 1G).

Based on these findings, we conclude that the peroxisomal proteins import was affected in HGPS fibroblasts, which may further contribute to their premature senescent phenotype.

### Catalase deficiency in HGPS fibroblasts

**Figure 2:**
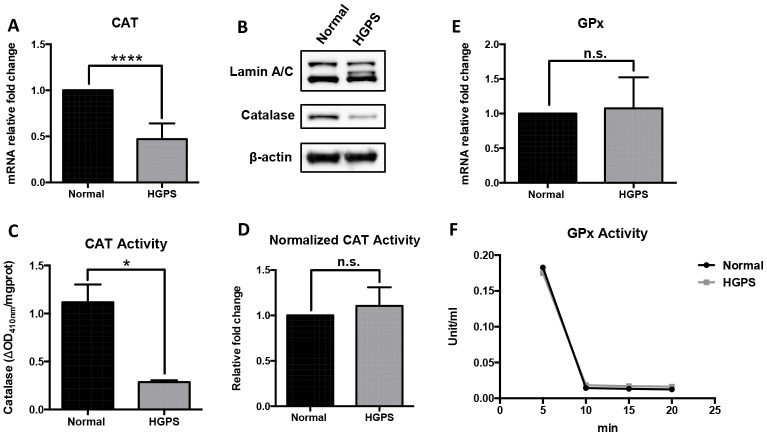
**Characterization of the ROS-scavenging enzymes in HGPS peroxisomes.** (**A**) Quantitative RT-PCR analysis of the relative expression of catalase in normal and HGPS fibroblasts. ****, p < 0.0001. (**B**) Western blot analysis of Lamin A/C, progerin and catalase expression in normal and HGPS fibroblasts at passage 25. (**C**) Catalase activity assay measured by the decomposed H_2_O_2_ per mg total protein from normal and HGPS fibroblasts. *, p < 0.05. (**D**) Catalase activity normalized with the protein expression level in normal and HGPS fibroblasts. n.s., not significant. (**E**) Quantitative RT-PCR analysis of the relative expression of Glutathione peroxidase in normal and HGPS fibroblasts. n.s., not significant. (**F**) Glutathione peroxidase activity in normal and HGPS fibroblasts. All experiments were performed using mid-passage cells between p15 to p25. All experiments were repeated at least three times and representative data were shown as indicated.

Since elevated oxidative stress was a hallmark in HGPS cells [9], we then investigated the behaviors of two major ROS scavenging enzymes in peroxisomes, namely catalase (CAT) and glutathione peroxidase (GPx) [28]. We detected a significant reduction in catalase mRNA transcription and protein expression in HGPS fibroblasts (Figure 2A and 2B). The overall catalase activity also showed a significant drop but turned back to a similar level as normal cells after normalizing with its protein abundance (Figure 2C and 2D). Contrarily, the relative expression and enzymatic activity of GPx family were not affected in HGPS cells (Figure 2E and 2F). In conclusion, our findings suggested that the inefficient ROS clearance in HGPS fibroblasts was at least partially due to a massive catalase deficiency.

### Exogenous progerin induced ROS production while down-regulating catalase expression in fibroblast cells

**Figure 3:**
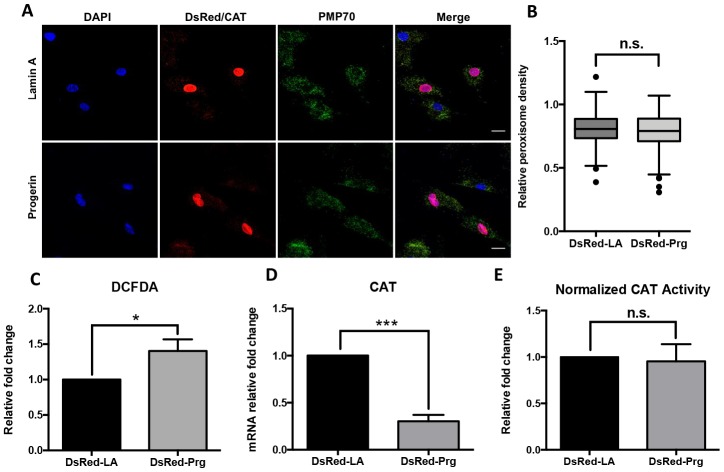
**Peroxisomal defects in normal fibroblasts expressing progerin.** (**A**) Normal human dermal fibroblast cells were infected by DsRed-LA and DsRed-Pg lentiviruses. The exogenous Lamin A and progerin expression were detected by DsRed fluorescence in the nuclei. Peroxisomes localization was indicated by PMP70 and catalase immunofluorescence staining. Bar = 25μm. (**B**) Quantification of PMP70 puncta per square unit in fibroblasts overexpressing lamin A and progerin. More than 200 cells from 3 independent experiments were analyzed and the data was represented in Tukey box plot. Boxes show the 25^th^, 50^th^, and 75^th^ percentiles and the dots indicate the outliers. (**C**) Relative fold change of ROS activity measured by DCFDA flow cytometry analysis in fibroblasts overexpressing lamin A and progerin. *, p < 0.05. (**D**) Quantitative RT-PCR analysis of the relative expression of catalase in fibroblasts overexpressing lamin A and progerin. ***, p < 0.001. (**E**) Normalized catalase activity in fibroblasts overexpressing lamin A and progerin. n.s., not significant. All experiments were performed using mid-passage cells between p15 to p25. All experiments were repeated at least three times and representative data were shown as indicated.

We further questioned if the catalase deficiency in HGPS fibroblasts was a downstream effect of progerin expression. To address that, human dermal fibroblasts (HGFDFN168) were transduced with lentiviral vectors expressing DsRed-tagged lamin A (DsRed-LA) and progerin (DsRed-Prg). We then identified the DsRed-positive cells and detected the peroxisomes distribution within these cells by confocal fluorescence microscopy (Figure 3A). The peroxisome density in DsRed-Prg positive cells turned out to be similar to the DsRed-LA positive control (Figure 3B). Consistent with Figure 1, no significant difference in GPx, Pex5 and Pex7 expression was detected (Supplementary Figure 2A and 2B). At three weeks post-viral infection, we detected a higher ROS level in cells with DsRed-Prg expression by 2’, 7’-dichlorofluorescein diacetate (DCFDA) flow cytometry assay (Figure 3C). However, there was a remarkable reduction of catalase transcription in DsRed-Prg expressing cells revealed by quantitative RT-PCR (Figure 3D). We did not detect an obvious reduction in catalase protein level in this experiment (Supplementary Figure 2A), likely due to the small portion of DsRed positive cells remained in the population three weeks after virus transduction. Consistently, the normalized catalase activity was unaffected in cells with exogenous progerin expression (Figure 3E). These findings suggested that the accumulation of exogenous progerin in the cell could cause excessive ROS production as well as reduced catalase expression.

### Catalase overexpression alleviates the oxidative stress in HGPS fibroblasts

**Figure 4:**
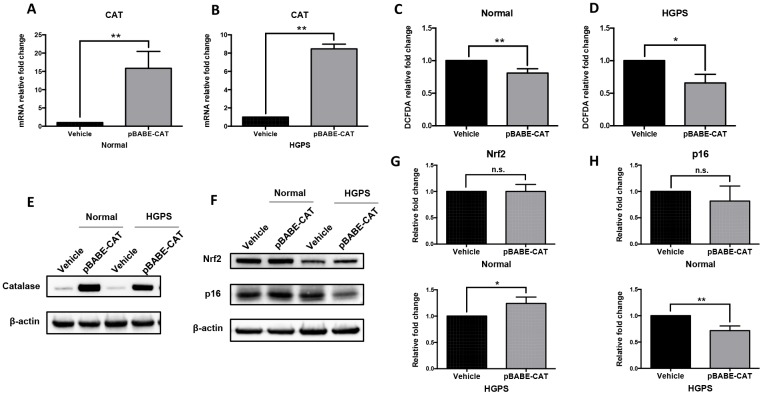
**Catalase overexpression alleviates oxidative stress in normal and HGPS fibroblasts.** (**A**, **B**) Quantitative RT-PCR analysis showed the relative expression of catalase in normal and HGPS fibroblasts infected by pBABE-CAT and control pBABE-puro retroviral vectors (Vehicle), respectively. **, p < 0.01. (**C**, **D**) Relative fold change of ROS activity measured by DCFDA flow cytometry analysis in normal and HGPS fibroblasts overexpressing catalase. *, p < 0.05, **, p < 0.01. (**E**) Western blot analysis showed catalase expression in normal and HGPS fibroblasts infected with pBABE-CAT and control vectors (cell passage number = 18). (**F**) Western blot analysis showed Nrf2 and p16 expression in normal and HGPS fibroblasts infected with pBABE-CAT and control vectors (cell passage number = 18). (**G**, **H**) Quantification of Nrf2 and p16 relative expression from Figure 4F. *, p < 0.05, **, p < 0.01, n.s., not significant. All experiments were performed using mid-passage cells between p15 to p25. All experiments were repeated at least three times and representative data were shown as indicated.

We then tested if catalase overexpression can reverse some of the aging hallmarks in normal and HGPS fibroblasts. We transduced the normal and HGPS fibroblast cells with recombinant human catalase-encoding retroviral vector (pBABE-CAT) and controlling pBABE-puro vector (vehicle). The catalase overexpression in pBABE-CAT infected cells was confirmed by quantitative RT-PCR (Figure 4A and 4B), Western blot (Figure 4E) and immunofluorescence staining (Supplementary Figure 3A) in both normal and HGPS fibroblasts. The compensated catalase expression resulted in a significant reduction of the cellular ROS level (Figure 4C and 4D). Again, no significant difference in Pex5 expression was observed (Supplementary Figure 3B and 3C).

We then detected the expression level of nuclear factor erythroid 2-related factor 2 (Nrf2) and p16 in fibroblasts with catalase overexpression. Nrf2 is a master regulator of the cellular antioxidant defense system [29]. In HGPS cells, the Nrf2 pathway was suppressed possibly due to the interference of progerin [11]. p16 is a biomarker of cellular senescence and skin aging [30]. We found that although in normal fibroblasts these proteins expression level remained unchanged, in HGPS cells overexpressing catalase, Nrf2 expression was restored moderately and the p16 expression was down-regulated compared to cells transduced by vehicle (Figure 4F–4H). To conclude, our findings suggested that catalase overexpression could alleviate the oxidative stress in HGPS fibroblasts.

### RAD001 reduces cellular ROS and stimulates catalase activity

**Figure 5:**
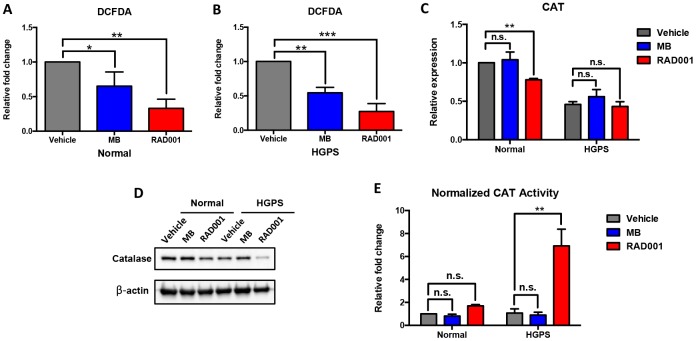
**Methylene Blue (MB) and RAD001 reduce cellular ROS and RAD001 activated catalase activity in HGPS cells.** Normal and HGPS fibroblasts were treated with 100nM Methylene Blue and RAD001 for 2 weeks. (**A**, **B**) The relative fold change of ROS activity was measured by DCFDA flow cytometry analysis. One-way ANOVA followed by Dunnett's multiple comparisons test was used to compare the effect of Methylene Blue and RAD001 treatment with the control group (Vehicle). *, p < 0.05, **, p < 0.01, ***, p < 0.001. (**C**) Relative mRNA expression of catalase was detected by quantitative RT-PCR analysis. Two-way ANOVA followed by Dunnett's multiple comparisons test was used to compare the mRNA expression of Methylene Blue and RAD001 treated cells with the control group within each block (normal and HGPS). n.s., not significant, **, p < 0.01. (**D**) Catalase expression level was detected by Western blot analysis (cell passage number = 19). (**E**) Normalized catalase activity. Two-way ANOVA followed by Dunnett's multiple comparisons test was used to compare the catalase activity of Methylene Blue and RAD001 treated cells with the control group within each block (normal and HGPS). n.s., not significant, **, p < 0.01. All experiments were repeated at least three times and representative data were shown as indicated.

We treated both normal and HGPS fibroblasts with 100 nM MB and RAD001 for two weeks, as described previously [9, 20]. We detected the ROS clearance effect of both treatments by DCFDA flow cytometry assay and found that both treatments were able to reduce ROS and RAD001 treatment appeared to be more effective than MB (Figure 5A and 5B). Furthermore, we analyzed the mRNA transcription, protein expression and enzymatic activity of catalase after drug treatment. Our findings indicated that the administrated MB treatment did not affect catalase expression (Figure 5C and 5D). There was a reduction of catalase mRNA expression in the normal cells after RAD001 treatment (Figure 5C), and at the protein level, catalase expression seems to be drastically reduced in HGPS (Figure 5D and quantification in Supplementary Figure 4A). However, very interestingly, when normalized with the protein amount, the unit catalase activity seems greatly enhanced in RAD001-treated HGPS cells (Figure 5E and Supplementary Figure 4B). Our findings suggested RAD001 alleviated ROS through stimulation of catalase activity.

## DISCUSSION

Peroxisomes targeting (especially PTS1 containing) proteins import have been found compromised in aging cells [16]. A recent study revealed that the PTS1 transporter Pex5 is a redox-sensitive protein and that the ROS-inactivated monoubiquitinylation at Pex5 Cys-11 may affect its PTS1 binding affinity [31, 32]. It is reasonable to infer that the interactions between Pex5 and PTS1-containing proteins weaken during normal aging due to the ROS accumulation in the cellular environment. Similarly, elevated oxidative stress may contribute to the moderately impaired peroxisomal protein trafficking we observed in HGPS fibroblasts.

The mammalian catalase contains a noncanonical PTS1 (-KANL), which has a relatively low binding affinity to the Pex5 receptor [26]. Previous studies indicated that in cells under oxidative stress, an increasing portion of catalase was retained in the cytosol, which may serve as an antioxidative defense system [32, 33]. In this study, we did not observe significantly mislocalized catalase in mid-passage HGPS fibroblasts and cells overexpressing progerin. Instead, we discovered a catalase deficiency that may be responsible for the ineffective ROS clearance in HGPS cells. Interestingly, humans with inherited catalase deficiency (or acatalasemia, hypocatalasemia) tend to have a high risk of developing age-related disorders including diabetes, atherosclerosis, and cancer [34, 35]. Thus, the down-regulated catalase expression in HGPS cells may be a downstream effect of progerin that further contributes to multiple premature aging phenotypes. Moreover, we found that catalase overexpression in HGPS fibroblasts restored the Nrf2 antioxidant pathway suppressed by progerin [11], and significantly improved the cellular redox homeostasis.

As a promising HGPS treatment, RAD001 promotes the progerin clearance by activating the autophagy machinery in HGPS cells [18, 20]. We observed a reduction of catalase expression in HGPS fibroblasts after RAD001 treatment, which may be regulated by the altered mTOR pathway. Surprisingly, our findings suggested that despite the overall reduction in catalase, RAD001 treatment enhances catalase unit activity in HGPS cells. On the contrary, MB, a mitochondrial-targeting antioxidant, significantly reduced cellular ROS but did not appear to affect either catalase activity or expression in HGPS. Our findings revealed a new role of RAD001 treatment in stimulating the catalase activity in HGPS.

In summary, we reported a series of peroxisomal abnormalities in HGPS dermal fibroblasts, including impaired proteins import, catalase deficiency, and ineffective ROS clearance. We found a peroxisomal effect of RAD001 treatment in HGPS cells that improves the catalase activity. In comparison, MB treatment appears to have less peroxisomal perturbations in HGPS cells. Future work needs to be done to investigate the peroxisomal movement in HGPS cells.

## MATERIALS AND METHODS

### Cell culture and drug treatment

The normal and HGPS human dermal fibroblast lines HGFDFN168 and HGFDFN167 were obtained from Progeria Research Foundation (PRF). HGFDFN167 carries the canonical *LMNA* c.1824C>T mutation of HGPS. All fibroblast cell lines were cultured in EMEM (Lonza, Basel, Switzerland) supplemented with 15% FBS (VWR International, Radnor, PA) at 37 °C with 5% CO^2^. Methylene Blue (MB; Acros Organics) and RAD001 (Everolimus; AdooQ BioScience) were dissolved in PBS and added to the growth medium at a final concentration of 100 nM. Fresh medium was provided twice a week, and the cells were passaged at ratio 1:3 when reaching 95% confluency. All the biomolecular detections in this work were performed using mid-passage cells between p15 to p25.

### Immunocytochemistry and peroxisomal quantification

Immunostaining was carried out using the following antibodies: catalase (Cell Signaling Technology, Danvers, MA) and PMP70 (Santa Cruz Biotechnology, Inc., Dallas, TX). DAPI (Vector Laboratories, Burlingame, CA, USA) was used to counterstain the cell nuclei. Images were acquired with Zeiss LSM 710 confocal microscope (Zeiss International, Oberkochen, Germany). Fluorescence intensity was adjusted with ImageJ software (NIH). Quantitative measurement of peroxisomes number per square unit was performed using ImageJ. In each green-fluorescence channel image, cells of interest were outlined and their surface areas (in pixels) were measured. These outlined cells were then analyzed using the particle analysis function for the number of particles greater than 4 × 4 pixels, which indicated the PMP70-positive peroxisomes. To account for the size difference of the cells, the peroxisomes counts were normalized with the cell surface areas.

### GFP-SCP2/PMP70 colocalization analysis

Normal and HGPS fibroblasts were transfected with GFP-SCP2 plasmid (a kind gift from Dr. Ralf Erdmann, RUB, Germany) using Lipofectamine 2000 (Thermo Fisher Scientific, Waltham, MA). At 48 hours post transfection, cells were fixed and immunostained with PMP70 antibody. Zeiss RGB fluorescence images were separated into respective channels in CellProfiler and named accordingly. The peroxisomes were identified and segmented by a primary object identification module, whereas cell borders were manually defined with the manual free drawing object module. Peroxisomes intensity was constrained using Otsu threshold and segmented by intensity automatic settings. A relate object module was implemented to quantify the percentage of PMP70 puncta not colocalized with GFP-SCP2.

### RNA isolation and quantitative real-time PCR

Total genomic RNA was extracted with Trizol (Life Technologies, Carlsbad, CA) and purified using the RNeasy Mini kit (Qiagen, USA) as per the manufacturer's instructions. The RNA yield was determined by the NanoDrop 2000 spectrophotometer (Thermo Fisher). 1 μg of total RNA was converted to cDNA using iScript Select cDNA Synthesis kit (Bio-Rad). Quantitative RT-PCR was performed in triplicate using SYBR Green Supermix (Bio-Rad) on CFX96 Real-Time PCR Detection System (C1000 Thermal Cycler, Bio-Rad). All primers used in this study are listed as follows: PEX5-Forward: 5′-CTGAATTCCTGCAGGACCAGAATGCA-3′; PEX5-Reverse: 5′-GTTCCCTCAGGTTCTCCCAGCCA-3′; PEX7-Forward: 5′-TTGATGTGACTTGGAGTGAGAA C-3′; PEX7-Reverse: 5′-CCCACAATTTGACAGTTTG ATCC-3′; CAT-Forward: 5′-TGGAGCTGGTAACCCA GTAGG-3′; CAT-Reverse: 5′-CCTTTGCCTTGGAGTA TTTGGTA-3′; GPx-Forward: 5′-AGATGAACGAGCT GCAGCGGCG-3′; GPx-Reverse: 5′-ACCGGAGACCA GGTGATGAGCTTG-3′.

### Western blotting assay

Whole cell lysates for immunoblotting were prepared by dissolving cell pellets in Laemmli Sample Buffer containing 5% 2-mercaptoethanol (Bio-Rad). Antibodies used in this study are as follows: lamin A/C (MAB3211; MilliporeSigma, Burlington, MA), catalase (Cell Signaling Technology, Danvers, MA), Nrf2 (sc-722, Santa Cruz), p16 (sc-468; Santa Cruz), and β-actin (A3854; Sigma-Aldrich, USA).

### Catalase activity and glutathione peroxidase activity assay

Cells grown on 100-mm culture dishes were trypsinized at 90% confluency, pelleted, and analyzed using the OxiSelect™ Catalase Activity Assay Kit, Fluorometric (STA-339, Cell Biolabs, Inc., San Diego, CA) and the Glutathione Peroxidase Assay Kit (ab102530; Abcam, Cambridge, United Kingdom) as per the manufacturer’s instructions. All “Normalized Catalase activity” data are normalized using Western blot quantification results.

### ROS measurement by DCFDA assay

To measure cellular ROS activity, cells on 100 mm dishes were trypsinized at 90% confluency, rinsed with PBS, and then incubated in 1× dilution buffer containing 12.5 μM DCFDA (ab113851; Abcam) at 37 °C. After 30 minutes incubation, the DCFDA intensity was detected by flow cytometry assay excited by a laser at 488 nm, and the data were recorded at 530 ± 15 nm. Flow cytometry was performed with FACS CantoII (BD Biosciences, San Jose, CA). The data were analyzed by FlowJo software (Ashland, OR, USA).

### Retroviral vectors production and virus transduction

Restriction enzymes SnabI and SalI (New England Biolabs, Ipswich, MA) were used to digest the human catalase sequence from the CAT-pC1 plasmid [36], which was then subcloned into the pBABE-puro retroviral vectors (a gift from Hartmut Land and Jay Morgenstern and Bob Weinberg, Addgene plasmid # 1764). After verification by sequencing, these retroviral constructs were co-transfected into HEK293T cells with two packaging vectors VSV-G, and Gag-Pol using Fugene 6 (Promega, E2692, Madison, WI). Two days after transfection, the culture supernatant containing viruses was purified by filtration through 0.45 μm filters and stored at -80 °C. Puromycin selection was performed after virus transduction at 1 μg/ml in culture media for 3 days.

### Statistical analysis

Statistical analysis was performed using GraphPad Prism 6.0 software (GraphPad Software, La Jolla, CA). The data are shown as mean ± SEM. Student’s t-test (two-tailed) was performed for the comparisons between two groups except for Figure 1D, where a Kolmogorov-Smirnov test was utilized. One-way ANOVA followed by Dunnett's multiple comparisons test was used to compare the effect of Methylene Blue and RAD001 treatment with control group (Vehicle); Two-way ANOVA followed by Dunnett's multiple comparisons test was used to compare the mRNA expression of Methylene Blue and RAD001 treated cells with the control group within each block (normal and HGPS); Two-way ANOVA followed by Dunnett's multiple comparisons test was used to compare the catalase activity of Methylene Blue and RAD001 treated cells with the control group within each block (normal and HGPS). P < 0.05 was considered statistically significant. All experiments were repeated at least three times and representative data were shown as indicated.

## Supplementary Material

Supplementary Figures
