# Supplementary material for: Peroxisomal abnormalities and catalase deficiency in Hutchinson-Gilford Progeria Syndrome
Source: Aging (Albany NY). 2020 Mar 18;12(6):5195–208. doi: 10.18632/aging.102941 (PMC7138560; doi:10.18632/aging.102941)
Supplement: Supplementary Figures [file aging-12-102941-s001..pdf]

SUPPLEMENTARY FIGURES

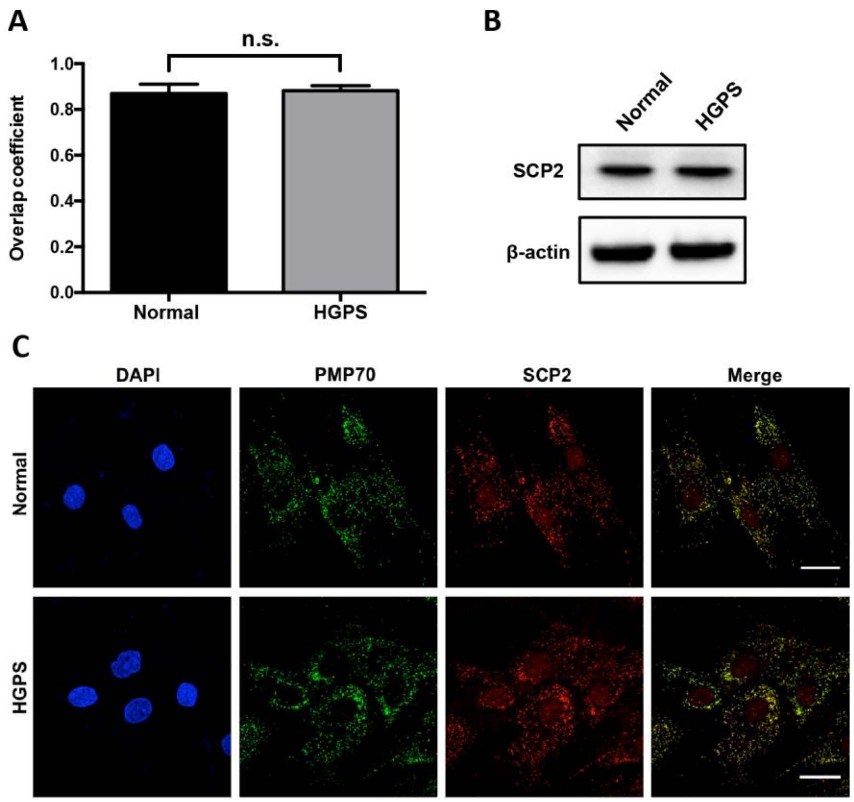

Supplementary Figure 1. (**A**) Colocalization of PMP70 and catalase fluorescence in normal and HGPS fibroblasts measured by a customized colocalization pipeline in CellProfiler. More than 100 cells from 3 independent experiments were analyzed. (**B**) Western blot analysis of endogenous SCP2 expression in normal and HGPS fibroblasts (cell passage number = 18). (**C**) Immunofluorescence staining of endogenous SCP2 in normal and HGPS fibroblasts. PMP70 antibody was used to indicate the peroxisomes localization. Bar = 25μm, n.s., not significant. All experiments were repeated at least three times and representative data were shown as indicated.

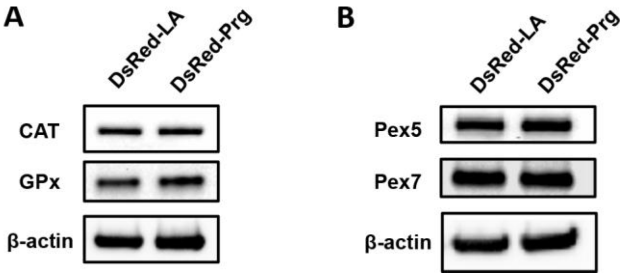

Supplementary Figure 2. (**A**, **B**) Western blot analysis of catalase, Glutathione peroxidase (GPx), Pex5 and Pex7 expression in DsRed-lamin A and DsRed-progerin expressing fibroblasts (cell passage number = 21). All experiments were repeated at least three times and representative data were shown as indicated.

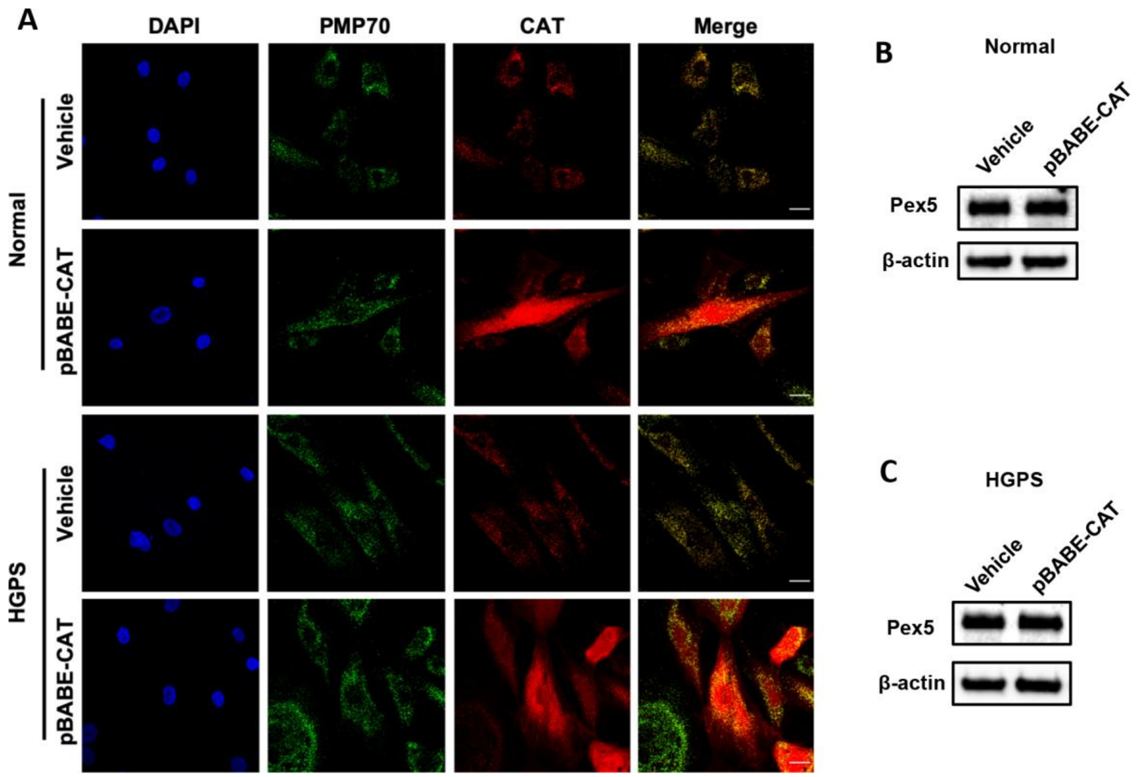

Supplementary Figure 3. (**A**) Immunofluorescence staining of catalase in normal and HGPS fibroblasts infected by pBABE-CAT and control vectors (Vehicle). PMP70 antibody was used to indicate the peroxisomes localization. Bar = 25µm. (**B**, **C**) Western blot analysis showed Pex5 expression in normal and HGPS fibroblasts infected by pBABE-CAT and Vehicle (cell passage number = 21). All experiments were repeated at least three times and representative data were shown as indicated.

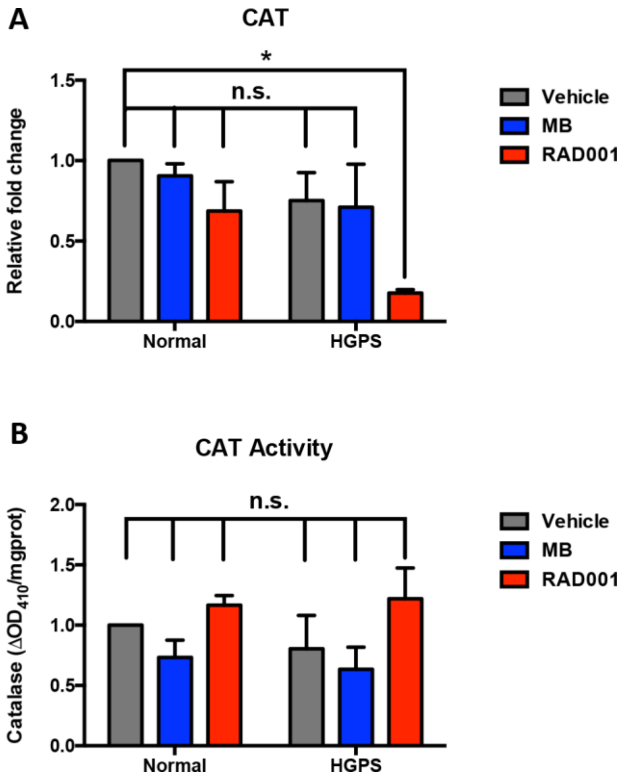

Supplementary Figure 4. (**A**) Quantification of catalase expression in normal and HGPS fibroblasts with MB and RAD001 treatment. (**B**) Raw catalase activity in normal and HGPS fibroblasts with MB and RAD001 treatment. *, $p < 0.05$, n.s., not significant. All experiments were repeated at least three times.
